# A Comparative Study of the Effects of Allantoin and Metformin on Blood Sugar Levels and Body Weight in Streptozotocin-Induced Diabetic Wistar Rats

**DOI:** 10.7759/cureus.111923

**Published:** 2026-07-01

**Authors:** Prathmesh R Gargate, Chitra Khanwelkar, Vandana M Thorat, Sujata Jadhav, Anjali Shinde, Kaushalendra Mani

**Affiliations:** 1 Department of Pharmacology, Krishna Institute of Medical Sciences, Krishna Vishwa Vidyapeeth (Deemed to be University), Karad, IND

**Keywords:** allantoin, body weight, diabetes mellitus, fasting blood sugar, fbs, herbal remedies, metformin, streptozotocin, t2dm, type 2 diabetes mellitus

## Abstract

Introduction

Diabetes is among the many known chronic diseases resulting from the inability of the body to produce insulin. The use of traditional drugs such as sulfonylurea, biguanide, and thiazolidinediones has been associated with some side effects, hence making researchers turn their attention toward herbal medicine as a treatment or prevention option for diabetes. Hence, the present study was designed to evaluate and compare the metabolic effects of allantoin (5 mg/kg and 10 mg/kg), metformin (500 mg/kg), and their combination on blood glucose and body weight in low-dose streptozotocin (STZ)-induced diabetic Wistar rats.

Methods

An in vivo study employed a randomized, controlled, parallel-group, preclinical experimental design consisting of a total of 36 young adult (8-10 weeks old) male Wistar rats (*Rattus norvegicus*) weighing 200-260 g, which were randomly assigned to six experimental groups, namely, G1 to G6. G1 and G2 served as non-diabetic and diabetic controls administered intraperitoneally (ip) with 2 ml of normal saline (NS) and STZ 45 mg/kg + 2 ml NS, respectively. G3, G4, G5, and G6 groups served as treatment groups and received STZ 45 mg/kg, concurrently administered intraperitoneally with allantoin (ALN) 5 mg/kg, ALN 10 mg/kg, metformin (MET) 500 mg/kg (oral), and ALN 5 mg/kg (ip) + MET 500 mg/kg (oral), respectively. Fasting blood sugar (FBS) estimation was done for nine days daily after STZ injection, after diagnosis of diabetes mellitus on the 9th/10th day, and on the 28th day of treatment. Body weight of experimental animals was recorded once weekly on days 0, 7, 14, 21, and 28.

Results

FBS levels differed significantly among groups at baseline and day 28 (p < 0.0001). Diabetic controls maintained persistently elevated glucose levels, whereas ALN treatment at 5 mg/kg (301.17 ± 11.18 vs. 218.33 ± 10.11; -82.84; p < 0.001) and ALN 10 mg/kg (302.83 ± 5.08 vs. 171.17 ± 7.88; -131.66; p < 0.001) significantly reduced FBS in a dose-dependent manner. Metformin monotherapy markedly improved glycemic control (297.00 ± 17.73 vs. 126.00 ± 10.10; -171.00; p < 0.001), while combination therapy with ALN and metformin produced the greatest reduction in glucose levels (302.50 ± 11.41 vs. 120.00 ± 9.57; -182.50; p < 0.001), suggesting additive antihyperglycemic effects. ALN 10 mg/kg significantly increased body weight (p = 0.002).

Conclusion

Allantoin, particularly along with metformin, offers promising therapeutic potential for the management of diabetes and associated metabolic alterations.

## Introduction

Diabetes mellitus is a widely recognized chronic metabolic disorder that results either from inadequate insulin production or the inability of body cells to effectively utilize insulin. The major forms of the disease include type 1 and type 2 diabetes mellitus (T2DM) [1]. Globally, diabetes affects over 537 million individuals, and this number is projected to increase to 783 million by the year 2045 [2]. Among the various forms, T2DM is encountered more frequently in clinical settings than type 1 diabetes mellitus [3]. Consequently, considerable attention has been directed toward the prevention and effective management of T2DM within healthcare systems. Current strategies for diabetes management involve multiple therapeutic approaches, such as dietary regulation, exercise, and pharmacological therapy [4]. Pharmacological treatment of T2DM presently includes insulin therapy and several classes of antihyperglycemic agents such as sulfonylureas, biguanides, and thiazolidinediones [5]. In recent years, herbal medicines have also emerged as promising alternatives for T2DM management because of their relatively fewer adverse effects and prolonged moderate therapeutic benefits [6].

Insulin resistance (IR) is considered the principal underlying pathological mechanism and an independent risk factor for T2DM [7]. This condition is characterized by diminished hepatic glycogen storage and decreased GLUT-4 expression in insulin-responsive cells [8]. Hepatic glycogen is essential for maintaining blood glucose balance, whereas GLUT-4 transporters are responsible for facilitating glucose uptake from the circulation into peripheral tissues [9]. IR develops as a result of abnormal insulin activity and disturbances in the PI3K/Akt signaling pathway, which regulates glucose transport, glycogen synthesis, glycolysis, and protein synthesis [10]. Therefore, improving IR remains a key therapeutic target in the management of T2DM. Several oral antihyperglycemic drugs, including metformin, rosiglitazone, and acarbose, are currently available for clinical use. However, these synthetic medications are often associated with undesirable adverse effects, highlighting the need to identify safer and more effective antidiabetic agents [11].

Herbal medicine has an important role to play in the prevention and cure of diabetes mellitus. Herbal products have several modes of action, including increased insulin secretion, improved insulin sensitivity, inhibition of carbohydrate-digesting enzymes, and reduction of oxidative stress. Due to this, there has been an upsurge in the research on herbal medicine and plant-based drugs for diabetes [2]. Allantoin, chemically known as 5-ureidohydantoin or 5-ureidoacetolactam, is a glyoxylic acid-derived diureide found abundantly in several plant sources, including yam, beetroot, maize, and wheat sprouts [12]. This purine-derived compound is considered safe and has not been associated with significant toxic effects. Allantoin possesses several pharmacological properties, including antioxidant, anti-inflammatory, wound-healing, and hypoglycemic activities. Previous studies have suggested the antidiabetic potential of both yam extract and allantoin [11]. In addition, investigations have explored their possible therapeutic benefits in diabetes management [13].

Allantoin (a diureide of glyoxylic acid) is a prominent constituent of yam extract. Interestingly, while allantoin is a normal end-product of purine metabolism in many mammals via the enzyme uricase, humans lack this enzyme, making uric acid the terminal metabolite [14,15]. However, allantoin is detectable in human serum and urine under conditions of increased oxidative stress, such as in diabetes and hypertension, suggesting a potential role as a marker or even a modulator in these pathological states [16,17].

Beyond its systemic presence, allantoin is widely recognized and approved for topical application, where it is valued for its anti-inflammatory, moisturizing, and wound-healing properties in dermatological and cosmetic formulations [18,19]. Despite its established topical safety and its natural occurrence in a plant with anti-diabetic folklore, the systemic pharmacological effects of allantoin, particularly concerning glucose and lipid metabolism, remain insufficiently explored.

For this reason, the current study was conducted to fill the gaps in the literature using a systematic comparison. Even though the crude extract of yam has been studied earlier, there is still a need for focused studies involving allantoin that will provide information on the mechanism of action of the antidiabetic property of the drug. In addition, no previous study has compared the effectiveness of allantoin with a well-known antidiabetic drug like metformin or the synergism between the two drugs. For this purpose, the current study was carried out using a streptozotocin-induced diabetic rat model to determine and compare the antidiabetic efficacy of two different doses of allantoin (5 mg/kg and 10 mg/kg), metformin (500 mg/kg), and the combination therapy of allantoin with metformin.

## Materials and methods

Study design

This study employed a randomized, controlled, parallel-group, preclinical experimental design at the Department of Pharmacology, Krishna Institute of Medical Sciences (KIMS), Krishna Vishwa Vidyapeeth (KVV), Karad, Maharashtra.

Animal selection and housing

A total of 36 young adult (8-10 weeks old) male Wistar rats (*Rattus norvegicus*) weighing 200-260 g were housed under standard conditions at the Central Animal House, KIMS, Karad. The temperature of the housing facility was maintained at 22.0 ± 2.0°C, and a 12-hour light/dark cycle (lights on at 8:00 am) was provided. Standard pellet diet and water ad libitum were given. The animals were acclimatized to laboratory conditions for one week before the experiment. All the experimental animals were assigned unique identification numbers. A computer-generated random number was provided to determine group allocation, and allocation was stratified by baseline body weight to ensure a balanced distribution. The allocation sequence was concealed until the interventions were assigned.

Group allocation

Six experimental groups, namely, G1-G6, were established. G1 and G2 served as non-diabetic and diabetic controls, administered intraperitoneally (ip) with 2 ml of normal saline (NS) and streptozotocin (STZ) 45 mg/kg + 2 ml NS, respectively. G3, G4, G5, and G6 groups served as treatment groups and received STZ 45 mg/kg, concurrently administered intraperitoneally with allantoin (ALN) 5 mg/kg, ALN 10 mg/kg, metformin (MET) 500 mg/kg (oral), and ALN 5 mg/kg (ip) + MET 500 mg/kg (oral), respectively. STZ was injected intraperitoneally (45 mg/kg), and fasting blood sugar (FBS) was tested every day for 9-10 days. After 9-10 days, rats with FBS ≥250 mg/dL were considered diabetic. All rats received a 28-day treatment with NS, ALN 5, ALN 10, MET 500, or ALN 5 + MET 500 as per the group treatment protocol. Drug administration was carried out between 9 am and 10 am every day.

Assessment parameters

FBS estimation was done for nine days daily after STZ injection, after diagnosis of T2DM on the 9th/10th day, and on the 28th day of treatment. Body weight of experimental animals was recorded once weekly on days 0, 7, 14, 21, and 28 after diagnosing the rat as diabetic.

Statistical analysis

Data entry and statistical analysis were carried out using Microsoft Excel 2021 (Microsoft Corporation, Redmond, WA) and SPSS version 28 (IBM Corp., Armonk, NY), respectively. The continuous variables were expressed in terms of mean and standard deviation (SD). Inferential statistics such as one-way ANOVA and Tukey’s honest significant difference (HSD) multiple comparison post-hoc test were performed. A paired t-test was used to analyze within-group data. P ≤ 0.05 was considered statistically significant.

## Results

Fasting blood sugar

Table 1 presents FBS level comparisons between study groups at different time intervals. FBS levels differed significantly among the study groups at baseline and on day 28 (ANOVA, p < 0.0001 for both), confirming successful induction of hyperglycemia and differential treatment responses. The normal control group (G1) maintained normoglycemic values throughout the study, with no significant change in FBS (p = 0.413). Similarly, the diabetic control group (G2) showed persistently elevated glucose levels with no significant reduction over the experimental period (p = 0.542), indicating sustained diabetic status. Treatment with ALN at both 5 mg/kg (G3) and 10 mg/kg (G4) produced significant reductions in FBS levels (p < 0.001), demonstrating dose-dependent antihyperglycemic activity. The higher dose of ALN showed greater glucose-lowering efficacy, with a mean reduction of 131.66 mg/dL compared to 82.84 mg/dL in the lower-dose group. Metformin monotherapy (G5) resulted in a marked decline in FBS levels, with values approaching near-normal levels by day 28. Notably, the combination therapy group (G6) exhibited the greatest reduction in FBS (182.50 mg/dL, p < 0.001). Overall, the results indicate that both ALN and metformin effectively ameliorated hyperglycemia in diabetic animals, with the combination regimen demonstrating superior glycemic control.

**Table 1:** Fasting blood sugar comparisons. ALN: allantoin; MET: metformin; G1: Group 1; G2: Group 2; G3: Group 3; G4: Group 4; G5: Group 5; G6: Group 6. *** Highly significant. Type of test statistics used: Data were expressed as mean ± SD (n = 6 per group). Within-group comparisons between day 0 and day 28 were performed using the paired Student's t-test. T-values and corresponding p-values are shown. Between-group comparisons at each time point were analyzed using one-way analysis of variance (ANOVA), followed by Tukey's post hoc multiple comparison test. F-values and corresponding p-values are presented at the bottom of the table. A p-value < 0.05 was considered statistically significant.

Group	Day 0	Day 28	Diff.	t-value	p-value
G1 - Normal control	113.50 ± 15.81	110.33 ± 10.50	-3.17	0.892	0.413
G2 - Diabetic control	298.33 ± 7.55	300.50 ± 10.77	2.17	-0.654	0.542
G3 - ALN 5 mg/kg	301.17 ± 11.18	218.33 ± 10.11	-82.84	18.234	<0.001^***^
G4 - ALN 10 mg/kg	302.83 ± 5.08	171.17 ± 7.88	-131.66	32.567	<0.001^***^
G5 - MET 500 mg/kg	297.00 ± 17.73	126.00 ± 10.10	-171.00	24.891	<0.001^***^
G6 - ALN 5 mg + MET 500 mg	302.50 ± 11.41	120.00 ± 9.57	-182.50	24.456	<0.001^***^
p-value	<0.0001	<0.0001			
F-value	232.41	334.46			

Body weight

The results of body weight comparisons at different time intervals are presented in Table 2. Baseline body weights differed significantly among the experimental groups (ANOVA, p = 0.0035), and this difference remained significant on day 28 (p = 0.0006), indicating variable treatment-related effects on body weight over the study period. The diabetic control group (G2) exhibited a significant reduction in body weight from day 0 to day 28 (p = 0.008), reflecting the catabolic state associated with diabetes induction. Similar significant decreases were observed in the ALN 5 mg/kg group (G3, p = 0.026) and metformin-treated group (G5, p = 0.012). In contrast, treatment with ALN 10 mg/kg (G4) produced a significant increase in body weight, with a mean gain of 18.19 g (p = 0.002), suggesting a potential protective or restorative metabolic effect at the higher dose. The normal control group (G1) and combination therapy group (G6) showed non-significant reductions in body weight (p > 0.05), indicating relative stabilization of body mass. Overall, the findings suggest that higher-dose ALN treatment was more effective in preventing diabetes-associated weight loss compared to low-dose ALN, metformin monotherapy, or combination treatment.

**Table 2:** Body weight comparison. ALN: allantoin; MET: metformin; G1: Group 1; G2: Group 2; G3: Group 3; G4: Group 4; G5: Group 5; G6: Group 6. * Significant. Type of test statistics used: Data were presented as mean ± SD (n = 6 per group). Within-group comparisons between day 0 and day 28 were performed using the paired Student's t-test, and the corresponding t-values and p-values are shown. Between-group comparisons at each time point were analyzed using one-way analysis of variance (ANOVA), followed by Tukey's post hoc multiple comparison test. Corresponding F-values and p-values are presented at the bottom of the table. A p-value < 0.05 was considered statistically significant.

Group	Day 0	Day 28	Diff.	t-value	p-value
G1 - Normal control	244.95 ± 14.05	236.58 ± 13.52	-8.37	2.156	0.084
G2 - Diabetic control	224.75 ± 10.51	217.07 ± 10.42	-7.68	4.231	0.008^*^
G3 - ALN 5 mg/kg	219.28 ± 8.38	212.33 ± 6.52	-6.95	3.112	0.026^*^
G4 - ALN 10 mg/kg	225.08 ± 8.23	243.27 ± 11.90	18.19	-5.876	0.002^*^
G5 - MET 500 mg/kg	237.08 ± 13.64	225.88 ± 16.27	-11.20	3.845	0.012^*^
G6 - ALN 5 mg + MET 500 mg	235.23 ± 10.26	228.77 ± 8.08	-6.46	2.012	0.100
p-value	0.0035	0.0006			
F-value	4.502	6.025			

## Discussion

Diabetes mellitus is commonly associated not only with persistent hyperglycemia but also with disturbances in body weight, both of which contribute substantially to chronic complications. Although metformin remains an effective agent for glycemic control, there is continuing interest in identifying compounds with wider metabolic benefits. Allantoin, a naturally occurring purine metabolite and an active constituent of Dioscorea batatas, has demonstrated promising antihyperglycemic activity in experimental studies [20,21]. Nevertheless, evidence directly comparing the efficacy of allantoin with metformin, as well as evaluating their combined effects, is still scarce. Therefore, the present study was undertaken to investigate and compare the effects of allantoin (5 mg/kg and 10 mg/kg), metformin (500 mg/kg), and their combination on fasting blood glucose and body weight in low-dose STZ-induced diabetic Wistar rats.

In the present investigation, diabetes was induced using a low-dose STZ model, which is extensively employed in experimental diabetes research involving rodents. STZ, a nitrosourea derivative, selectively damages pancreatic β-cells because of its preferential uptake through the GLUT2 glucose transporter [22]. Following cellular uptake, STZ induces DNA alkylation, activation of poly ADP-ribose polymerase (PARP), nitric oxide production, and oxidative stress, ultimately leading to β-cell dysfunction and partial destruction [23]. Administration of low-dose STZ, particularly in animals receiving a high-fat diet, produces moderate insulin deficiency accompanied by insulin resistance, thereby mimicking important pathological characteristics of T2DM [24]. The model consistently produces sustained hyperglycemia along with alterations in body weight, making it appropriate for evaluating anti-hyperglycemic and metabolic effects of investigational compounds.

Fasting blood sugar comparisons

The findings of the present study demonstrated that, at baseline (day 0), the normal control group differed highly significantly from all diabetic groups, including the diabetic control, allantoin-treated groups (5 mg/kg and 10 mg/kg), metformin-treated group, and combination therapy group. This marked statistical difference confirms the successful induction of diabetes using STZ and establishes a clear metabolic distinction between normal and diabetic animals. In intra-group analysis, both the normal control and diabetic control groups exhibited no significant changes over the 28-day experimental period, indicating maintenance of normoglycemia and persistent hyperglycemia, respectively. Conversely, all treatment groups showed a highly significant reduction in FBS levels. Administration of allantoin at 5 mg/kg reduced FBS by 82.84 mg/dL, whereas the 10 mg/kg dose produced a greater reduction of 131.66 mg/dL. Metformin treatment resulted in a decrease of 171.00 mg/dL, while combination therapy achieved the greatest reduction of 182.50 mg/dL, lowering the mean fasting blood glucose level to 120.00 mg/dL. Inter-group ANOVA performed on day 28 demonstrated a highly significant difference among the groups, indicating differential glycemic responses following treatment.

These observations are in agreement with the findings of Zhao et al., who reported that allantoin enhanced peripheral glucose utilization and restored glycogen content in hepatic and muscular tissues, with superior improvement in insulin sensitivity compared to metformin [11]. The antihyperglycemic effect of allantoin may be attributed to several mechanisms, including (i) activation of imidazoline I3 receptors on pancreatic β-cells, thereby enhancing insulin secretion [25], and (ii) stimulation of the AMPK signaling pathway, which increases glucose uptake and suppresses hepatic gluconeogenesis [26].

Although metformin demonstrated potent antihyperglycemic activity, higher doses of allantoin exhibited comparable efficacy. Notably, combined administration of allantoin and metformin produced a greater glucose-lowering effect, suggesting complementary mechanisms of action, including suppression of hepatic glucose production by metformin together with enhancement of peripheral insulin sensitivity by allantoin. Additionally, the possible β-cell protective effect of allantoin may contribute to improved endogenous insulin availability. These findings support the therapeutic rationale for combination therapy targeting multiple metabolic abnormalities associated with T2DM [12,25].

The results obtained in the present study are also consistent with earlier experimental studies investigating the anti-hyperglycemic potential of allantoin. Niu et al. and Lin et al. similarly observed significant reductions in plasma glucose levels in STZ-induced diabetic rats treated with allantoin [20,27]. The dose-dependent glucose reduction identified in the current study is in accordance with the observations of Niu et al., who reported greater glycemic improvement with higher doses of allantoin [20]. Furthermore, Zhao et al. demonstrated improvement in FBS levels in high-fat diet plus STZ-induced diabetic models, further supporting the glucose-lowering potential of allantoin across different experimental models of diabetes [11]. Collectively, these findings strengthen the evidence supporting the antihyperglycemic efficacy of allantoin.

Body weight comparisons

The present study also demonstrated that there was no statistically significant difference in body weight among diabetic groups at baseline, indicating comparable pre-treatment conditions. During the 28-day study period, the diabetic control group showed a reduction in body weight, reflecting the catabolic state associated with uncontrolled diabetes. Changes in body weight in diabetic models generally reflect insulin deficiency, increased catabolism, and disturbances in energy homeostasis. In the current investigation, treatment with allantoin 5 mg/kg resulted in a decrease in body weight, whereas allantoin 10 mg/kg produced an increase in body weight relative to diabetic controls. This effect may be associated with improved glycemic control, reduction of glucotoxicity, modulation of appetite-regulating pathways through I1 receptor activation [28], and restoration of muscle mass [21]. Interestingly, while metformin is generally considered weight-neutral or mildly weight-reducing, allantoin appears to exert modulatory effects on adiposity and overall energy balance.

In the current study, high-dose allantoin significantly increased body weight, suggesting recovery from diabetes-associated catabolic wasting, whereas metformin and low-dose allantoin were associated with weight reduction. Comparable findings were reported by Semol et al., who observed preservation and improvement of body weight due to reduced muscle wasting in diabetic rats [29]. In contrast, studies conducted in obesity and high-fat diet models by Chung et al. [28] and Ma et al. [21] demonstrated significant reductions in body weight and adiposity following allantoin treatment. These findings suggest that allantoin may exert dual regulatory effects, reducing excess adiposity in obesity models while restoring body weight in severe diabetic conditions characterized by catabolic loss.

Clinical implications

The clinical relevance of the present study lies in the possibility that, if these findings are reproduced in human studies, allantoin could emerge as a useful adjunctive therapy for T2DM by providing broad metabolic benefits and serving as a cost-effective phytopharmacological intervention. However, several limitations of the present study should be considered, including the absence of insulin estimation, lack of HOMA-IR (homeostatic model assessment of insulin resistance) assessment, omission of oxidative stress biomarkers, relatively short study duration, and absence of histopathological evaluation of pancreatic, hepatic, and renal tissues. Future investigations should focus on detailed molecular pathway analysis involving AMPK, PPAR-α, SIRT1, and Nrf2 signaling, assessment of inflammatory cytokines, histopathological evaluation of target organs, long-term safety studies, and translational clinical research.

## Conclusions

In conclusion, the present study demonstrated that diabetes induction resulted in significant hyperglycemia and progressive body weight reduction in experimental animals, confirming the establishment of a diabetic state. Treatment with allantoin produced notable antihyperglycemic effects in a dose-dependent manner, with the 10 mg/kg dose showing superior efficacy compared to the 5 mg/kg dose. Although metformin monotherapy exhibited marked glucose-lowering activity, the combination of allantoin and metformin achieved the greatest reduction in FBS levels, suggesting a potential synergistic therapeutic effect. In terms of body weight, higher-dose allantoin treatment significantly improved body weight compared to diabetic controls, indicating possible beneficial effects on metabolic status and overall health. Collectively, these findings suggest that allantoin, particularly in combination with metformin, offers promising therapeutic potential for the management of diabetes and associated metabolic alterations.
